# Identification of species belonging to the *Bifidobacterium* genus by PCR-RFLP analysis of a *hsp60* gene fragment

**DOI:** 10.1186/1471-2180-13-149

**Published:** 2013-07-01

**Authors:** Loredana Baffoni, Verena Stenico, Erwin Strahsburger, Francesca Gaggìa, Diana Di Gioia, Monica Modesto, Paola Mattarelli, Bruno Biavati

**Affiliations:** 1 Department of Agricultural Sciences, University of Bologna, viale Fanin 42, 40127, Bologna, Italy; 2 Laboratorio de Microbiología Molecular y Biotecnología Ambiental, Departamento de Química and Center of Nanotechnology and Systems Biology, Universidad Técnica Federico Santa María, Avenida España 1680, Valparaíso, Chile

**Keywords:** *Bifidobacterium* spp, *hsp60*, PCR-RFLP, Taxonomy

## Abstract

**Background:**

*Bifidobacterium* represents one of the largest genus within the *Actinobacteria*, and includes at present 32 species. These species share a high sequence homology of 16S rDNA and several molecular techniques already applied to discriminate among them give ambiguous results.

The slightly higher variability of the *hsp60* gene sequences with respect to the 16S rRNA sequences offers better opportunities to design or develop molecular assays, allowing identification and differentiation of closely related species. *hsp60* can be considered an excellent additional marker for inferring the taxonomy of the members of *Bifidobacterium* genus.

**Results:**

This work illustrates a simple and cheap molecular tool for the identification of *Bifidobacterium* species. The *hsp60* universal primers were used in a simple PCR procedure for the direct amplification of 590 bp of the *hsp60* sequence. The *in silico* restriction analysis of bifidobacterial *hsp60* partial sequences allowed the identification of a single endonuclease (HaeIII) able to provide different PCR-restriction fragment length polymorphism (RFLP) patterns in the *Bifidobacterium* spp. type strains evaluated. The electrophoretic analyses allowed to confirm the different RFLP patterns.

**Conclusions:**

The developed PCR-RFLP technique resulted in efficient discrimination of the tested species and subspecies and allowed the construction of a dichotomous key in order to differentiate the most widely distributed *Bifidobacterium* species as well as the subspecies belonging to *B. pseudolongum* and *B. animalis*.

## Background

Members of the genus *Bifidobacterium* are Gram-positive, obligate anaerobic, non-motile, non-spore forming bacteria [1], and are the most important constituents of human and animal intestinal microbiota [2,3]. Recently, news species of bifidobacteria have been described [4-6] and now more than 30 species have been included in this genus.

*Bifidobacterium* spp. can be detected in various ecological environments, such as intestines of different vertebrates and invertebrates, dairy products, dental caries and sewage. Considering the increasing application of *Bifidobacterium* spp. as protective and probiotic cultures [7-9], and the fast enlargement of the genus, easy identification tools to discriminate new isolates are essential. Moreover, their correct taxonomic identification is of outmost importance for their use as probiotics [2]. Conventional identification and classification of *Bifidobacterium* species have been based on phenotypic and biochemical features, such as cell morphology, carbohydrate fermentation profiles, and polyacrylamide gel electrophoresis analysis of soluble cellular proteins [10]. In the last years several molecular techniques have been proposed in order to identify bifidobacteria. Most available bifidobacterial identification tools are based on 16S rRNA gene sequence analysis, such as ARDRA [11,12], DGGE [13] and PCR with the use of species-specific primers [14-16]. However, 16S rDNA of *Bifidobacterium* spp. has a high similarity, ranging from 87.7 to 99.5% and bifidobacterial closely related species (e.g. *B. catenulatum* and *B. pseudocatenulatum*) or subspecies (e.g. *B. longum* and *B. animalis* subspecies) even possess identical 16S rRNA gene sequences [17,18]. For this reason different molecular approaches have been tested based on repetitive genome sequences amplification, such as ERIC-PCR [19,20], BOX-PCR [21,22] or RAPD fingerprinting analysis [23]. These fingerprinting methods have the disadvantage of a low reproducibility, and they need strict standardization of PCR conditions. The use of different polymerases, DNA/primer ratios or different annealing temperatures may lead to a discrepancy in the results obtained in different laboratories [24].

In recent years alternative molecular markers have been proposed for bifidobacteria identification (e.g. *hsp60*, *recA*, *tuf*, *atpD*, *dnaK*) and Ventura et al. [18] developed a multilocus approach, based on sequencing results, for the analysis of bifidobacteria evolution. The *hsp60* gene, coding for a highly conserved 60 kDa heat-shock-protein (a chaperonin), has been evaluated for phylogenetic analysis in bifidobacteria by Jian et al. [25]. The sequence comparison of this gene has been already used for species identification and phylogenetic analysis of other genera (e.g. *Staphylococcus*, *Lactobacillus*) and enteric pathogens [26-28]. A chaperonin database (cpnDB) is available on line, collecting bacterial and eukaryotic sequences (http://www.cpndb.ca/cpnDB/home.php) [29].

The purpose of this study is the development of a rapid, reproducible and easy-to-handle molecular tool for the identification of *Bifidobacterium* species isolated from various environments. The protocol is based on the restriction endonuclease analysis of the PCR-amplified *hsp60* partial gene sequence (*hsp60* PCR-RFLP) with the use of a single restriction enzyme and has been tested on the 30 most widely distributed *Bifidobacterium* species and subspecies. A diagnostic dichotomous key to speed up profile interpretation has also been proposed.

## Methods

### Bacterial strains and culture conditions

The type strains used to develop the technique are listed in Table 1, whereas the strains used to validate the method are reported in Table 2. The strains, belonging to BUSCoB (Bologna University Scardovi Collection of Bifidobacteria) collection, were isolated from faeces of human and animals and from sewage. Bacteria were maintained as frozen stocks at −80°C in the presence of skim milk as cryoprotective agent. Working cultures were prepared in TPY medium [1], grown anaerobically at 37°C and harvested at logarithmic phase.

**Table 1:** Type-strains investigated

**Species**	**International culture collection**
*Bifidobacterium adolescentis*	ATCC 15703
*Bifidobacterium angulatum*	ATCC 27535
*Bifidobacterium animalis* subsp*. animalis*	ATCC 25527
*Bifidobacterium animalis* subsp*. lactis*	DSM 10140
*Bifidobacterium asteroides*	ATCC 25910
*Bifidobacterium bifidum*	ATCC 29521
*Bifidobacterium boum*	ATCC 27917
*Bifidobacterium breve*	ATCC 15700
*Bifidobacterium catenulatum*	ATCC 27539
*Bifidobacterium choerinum*	ATCC 27686
*Bifidobacterium coryneforme*	ATCC 25911
*Bifidobacterium cuniculi*	ATCC 27916
*Bifidobacterium dentium*	ATCC 27534
*Bifidobacterium gallicum*	ATCC 49850
*Bifidobacterium gallinarum*	ATCC 33777
*Bifidobacterium indicum*	ATCC 25912
*Bifidobacterium longum* subsp*. longum*	ATCC 15707
*Bifidobacterium longum* subsp*. infantis*	ATCC 15697
*Bifidobacterium longum* subsp*. suis*	ATCC 27533
*Bifidobacterium minimum*	ATCC 27539
*Bifidobacterium merycicum*	ATCC 49391
*Bifidobacterium pseudolongum* subsp *pseudolongum*	ATCC 25526
*Bifidobacterium pseudolongum* subsp*. globosum*	ATCC 25865
*Bifidobacterium pseudocatenulatum*	ATCC 27919
*Bifidobacterium pullorum*	ATCC 27685
*Bifidobacterium ruminantium*	ATCC 49390
*Bifidobacterium subtile*	ATCC 27537
*Bifidobacterium thermacidophilum* subsp. *porcinum*	LMG 21689
*Bifidobacterium thermacidophilum* subsp. *thermacidophilum*	LMG 21395
*Bifidobacterium thermophilum*	ATCC 25525

**Table 2:** List of strains investigated to confirm the conservation of RFLP profiles (strains belonging to BUSCoB collection)

Species^*^	**Strain**	**Source**
*Bifidobacterium animalis* subsp*. animalis*	T169	Rat
*Bifidobacterium animalis* subsp*. animalis*	T6/1	Rat
*Bifidobacterium animalis* subsp*. lactis*	P23	Chicken
*Bifidobacterium animalis* subsp*. lactis*	F439	Sewage
*Bifidobacterium animalis* subsp*. lactis*	Ra20	Rabbit
*Bifidobacterium animalis* subsp*. lactis*	Ra18	Rabbit
*Bifidobacterium animalis* subsp*. lactis*	P32	Chicken
*Bifidobacterium bifidum*	B1764	Infant
*Bifidobacterium bifidum*	B2091	Infant
*Bifidobacterium bifidum*	B7613	Preterm infant
*Bifidobacterium bifidum*	B2009	Infant
*Bifidobacterium bifidum*	B2531	Infant
*Bifidobacterium breve*	B2274	Infant
*Bifidobacterium breve*	B2150	Infant
*Bifidobacterium breve*	B8279	Preterm infant
*Bifidobacterium breve*	B8179	Preterm infant
*Bifidobacterium breve*	Re1	Infant
*Bifidobacterium catenulatum*	B1955	Infant
*Bifidobacterium catenulatum*	B684	Adult
*Bifidobacterium catenulatum*	B2120	Infant
*Bifidobacterium pseudocatenulatum*	B1286	Infant
*Bifidobacterium pseudocatenulatum*	B7003	
*Bifidobacterium pseudocatenulatum*	B8452	
*Bifidobacterium dentium*	Chz7	Chimpanzee
*Bifidobacterium dentium*	Chz15	Chimpanzee
*Bifidobacterium longum* subsp*.longum*	PCB133	Adult
*Bifidobacterium longum* subsp*. infantis*	B7740	Preterm infant
*Bifidobacterium longum* subsp*. infantis*	B7710	Preterm infant
*Bifidobacterium longum* subsp*. suis*	Su864	Piglet
*Bifidobacterium longum* subsp*. suis*	Su932	Piglet
*Bifidobacterium longum* subsp*. suis*	Su905	Piglet
*Bifidobacterium longum* subsp*. suis*	Su908	Piglet
*Bifidobacterium pseudolongum* subsp. *pseudolongum*	MB9	Chicken
*Bifidobacterium pseudolongum* subsp. *pseudolongum*	MB10	Mouse
*Bifidobacterium pseudolongum* subsp. *pseudolongum*	MB8	Chicken
*Bifidobacterium pseudolongum* subsp*. globosum*	Ra27	Rabbit
*Bifidobacterium pseudolongum* subsp*. globosum*	VT366	Calf
*Bifidobacterium pseudolongum* subsp*. globosum*	T19	Rat
*Bifidobacterium pseudolongum* subsp*. globosum*	P113	Chicken

^*^ previously assigned taxonomic identification.

### *In silico* analysis

An *in silico* analysis was performed for the evaluation of a suitable restriction enzyme. Available *hsp60* sequences had been retrieved from cpnDB database and GeneBank, thanks to the work of Jian et al. [25]. *In silico* digestion analysis was carried out on fragments amplified by universal primers H60F-H60R [30] using two on-line free software: webcutter 2.0 (http://rna.lundberg.gu.se/cutter2) and http://insilico.ehu.es/restriction softwares [31]. Blunt end, frequent cutter enzymes that recognize not degenerated sequences have been considered in order to find a suitable enzyme for all the species (e.g. RsaI, HaeIII, AluI, AccII). However *in silico* analysis had been performed also on sticky end enzymes (e.g. AatII, Sau3AI, PvuI).

### DNA extraction from pure cultures

10 ml of culture were harvested and washed twice with TE buffer (10 mM Tris–HCl, 1 mM EDTA, pH 7.6), resuspended in 1 ml TE containing 15 mg lysozyme and incubated at 37°C overnight. Cells were lysed with 3 ml of lysis buffer (100 mM Tris–HCl, 400 mM NaCl, 2 mM EDTA, pH 8.2), 220 μl SDS (10% w/v) and 150 μl proteinase K (>600 mAU/ml, solution) and incubated for 2 hours in water bath at 60°C. One ml of saturated NaCl solution was added and the suspension was gently inverted twice. Pellets were harvested through centrifugation (5000 × g) at room temperature for 15 minutes. After the transfer of clean supernatants in new tubes, DNA was precipitated with 2.5 volumes of cold ethanol (95%) and resuspended in 300 μl of TE buffer [32].

### Amplification of gene *hsp60* and restriction with HaeIII

Universal primers were used to amplify approximately 600 bp of the *hsp60* gene in the *Bifidobacterium* spp. investigated. These primers H60F (5‘-GG(ATGC)GA(CT)GG(ATGC)AC(ATGC)AC(ATGC)AC(ATGC)GC(ATGC)AC(ATGC)GT-3’) and H60R (5’-TC(ATGC)CC(AG)AA(ATGC)CC(ATGC)GG(ATGC)GC(CT)TT(ATGC)AC(ATGC)GC-3’) were designed by Rusanganwa et al. [30] on the basis of the conserved protein sequences GDGTTATV and AVKAPGFGD in HSP60. Amplifications were performed in 20 μl volumes with 1.5 μM of each primer (Eurofins MWG Operon, Ebersberg, Germany), 10 μl 2X HotStarTaq Plus Master Mix (Qiagen, Italy) (1,5 mM MgCl_2_, 1 U Taq, 0.2 mM dNTP, final concentration) and 150 ng/μl DNA. The PCR cycle consisted of an initial denaturation of 5 min at 95°C followed by 35 cycles of denaturation (30s at 94°C), annealing (30s at 61°C) and extension (45 s at 72°C). The PCR was completed with a final elongation of 10 min at 72°C. The PCR amplification was performed with a PCR Verity 96-well thermal cycler (Applied Biosystems, Milan, Italy). After amplification, the product was visualized via agarose gel (1.3% w/v) in 1X TBE buffer and visualized with ethidium bromide under UV light. A 100 bp DNA ladder (Sigma-Aldrich) was used as a DNA molecular weight marker. Bands were excised from agarose gel (Additional file 1: Figure S1) and DNA was eluted with NucleoSpin® Gel and PCR Clean-up (Macherey-Nagel GmbH & Co. KG, Germany) in order to avoid possible non-specific amplifications. 3 μl of the eluted DNA was re-amplified in a 30 μl PCR reaction (see above). BSA was added to the reaction (5% v/v, Fermentas). The PCR products (2 μl) were checked for non-specific amplification on agarose gel. 20 μl (~6 μg) of PCR amplicons were digested with HaeIII enzyme. Restriction digestion was carried out for 2 h at 37°C in 30 μl reaction mixture with 1X SM Restriction Buffer (Sigma-Aldrich), 1.5 μl HaeIII (10 U/μl, Sigma-Aldrich) and water. Digestion products were stained with ethidium bromide and visualized under UV-light (GelDoc™, BioRad), after agarose gel electrophoresis (3.0% agarose (w/v), TBE 1X) at 210 V (3 h). A 20 bp DNA ladder (Sigma-Aldrich) was used. The obtained pictures were elaborated with a free software GNU Image Manipulation Program (Gimp 2.8) only to invert colors and increase contrast.

Precast gradient polyacrylamide gels (4-20%) (Lonza Group Ltd, Switzerland) were also used to obtain RFLP profiles, in order to have a comparison with agarose gels. The vertical electrophoresis apparatus used was P8DS™ Emperor Penguin (Owl, Thermo Scientific) with an adaptor for Lonza precast gels. The run was performed at 100 V in TBE 1X.

### Diagnostic key

A dichotomous key was developed comparing *in silico* digestion results and the evaluation of visible bands with the use of ImageLab™ 2.0 software (Bio-Rad Laboratories, Inc.).

## Results and discussion

### *In silico* analysis

The analysis and comparison of restriction profiles obtained with *in silico* digestion of bifidobacterial *hsp60* sequences allowed the identification of a set of appropriate frequent-cutter endonucleases that recognize non degenerated sequences. The restriction enzyme HaeIII was found to give the clearest and most discriminatory profiles in theoretical PCR-RFLP patterns, discriminating the majority of *Bifidobacterium* type-strains tested (Table 3). Furthermore, the profiles of other strains, belonging to the investigated species, have been analyzed to confirm the conservation of RFLP profiles within species.

**Table 3:** **Expected fragment sizes obtained with *in silico *digestion of the *hsp60 *gene sequences**

***Bifidobacterium *species**	**GenBank entry**	**Predicted fragment sizes**	**Profile**
*B. adolescentis*	AF210319	31-36-81-103-339	
*B. angulatum*	AF240568	42-54-59-139-296	
*B. animalis* subsp*. animalis*	AY004273	17-53-86-97-114-223	
*B. animalis* subsp*. lactis*	AY004282	71-86-96-114-223	
*B. asteroides*	AF240570	30-38-75-97-109-242	
*B. bifidum*	AY004280	22-31-59-181-297	
*B. boum*	AY004285	22-117-200-251	
*B. breve*	AF240566	106-139-139-200	
*B. catenulatum*	AY004272	53-198-338	
*B. choerinum*	AY013247	36-42-51-52-54-59-97-200	
*B. coryneforme*	AY004275	16-32-54-158-338	
*B. cuniculi*	AY004283	16-42-53-70-128-281	
*B. dentium*	AF240572	22-31-42-68-130-139-158	
*B. gallicum*	AF240575	42-253-297	
*B. gallinarum*	AY004279	16-31-42-81-139-281	
*B. indicum*	AF240574	16-32-36-42-45-123-296	
*B. longum* subsp*. longum*	AF240578	42-113-138-139-158	*
*B. longum* subsp*. infantis*	AF240577	42-113-138-139-158	*
*B. longum* subsp*. suis*	AY013248	42-113-138-139-158	*
*B. merycicum*	AY004277	22-31-42-59-139-297	
*B. minimum*	AY004284	16-51-60-66-70-327	
*B. pseudocatenulatum*	AY004274	42-53-198-297	
*B. pseudolongum* subsp *pseudolongum*	AY004282	17-22-30-32-42-42-109-297	
*B. pseudolongum* subsp*. globosum*	AF286736	16-17-22-30-32-42-109-323	
*B. pullorum*	AY004278	16-31-36-42-81-87-297	
*B. ruminantium*	AF240571	31-106-114-339	
*B. subtile*	Not available	Not avaiable	+
*B. thermacidophilum* subsp *porcinum*	AY004276	20-42-53-59-97-139-180	*†
*B. thermacidophilum* subsp *thermacidophilum*	AY004276	20-42-53-59-97-139-180	*†
*B. thermophilum*	AF240567	54-59-117-139-222	

+ hsp60 sequence of *B. subtile* type strain was not available in the press-time.

† the available sequences at GeneBank and cpnDB belonged to *B. thermacidophilum* (with no distinction in subspecies).

*subspecies not discernable.

### Amplification and restriction analysis of *Bifidobacterium* spp

Theoretical restriction profiles have been confirmed *in vitro* on agarose gel. The obtained fragments ranged from 16 bp to 339 bp (Table 3). Fragments lower than 25 bp were not considered as they did not help in species discrimination and in addition they co-migrate with primers. Time course analysis of restricted samples showed the formation of a band of ~200 bp in several species due to an over-digestion (data not shown) and this invalidated the RFLP profiles. For this reason the protocol has been optimized at 2 hours restriction time. Fragments greater than 360 bp were also not considered due to a possible incomplete digestion of such long fragments.

**Figure 1:**
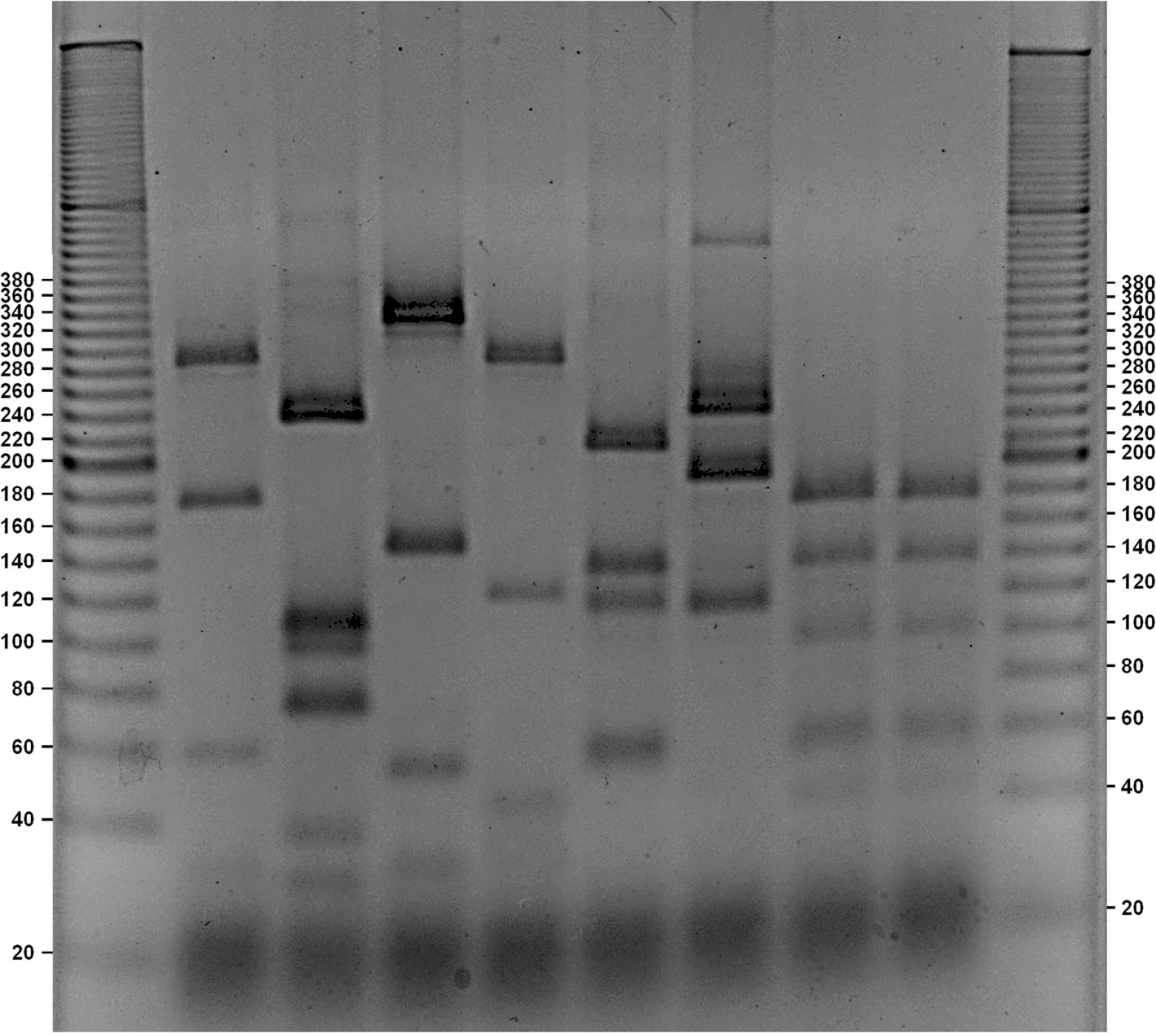
**Agarose gel electrophoresis of digested *hsp60 *DNA fragments with HaeIII (negative image).** Lane1, ladder 20 bp (Sigma-Aldrich); Lane 2, *B. bifidum* ATCC 29521; Lane 3, *B. asteroides* ATCC 25910, Lane 4, *B. coryneforme* ATCC 25911; Lane 5, *B. indicum* ATCC 25912; Lane 6, *B. thermophilum* ATCC 25525; Lane 7, *B. boum* ATCC 27917; Lane 8, *B. thermacidophilum* subsp. *porcinum* LMG 21689; Lane 9, *B. thermacidophilum* subsp. *thermacidophilum* LMG 21395; Lane 10, ladder 20 bp (Sigma-Aldrich).

**Figure 2:**
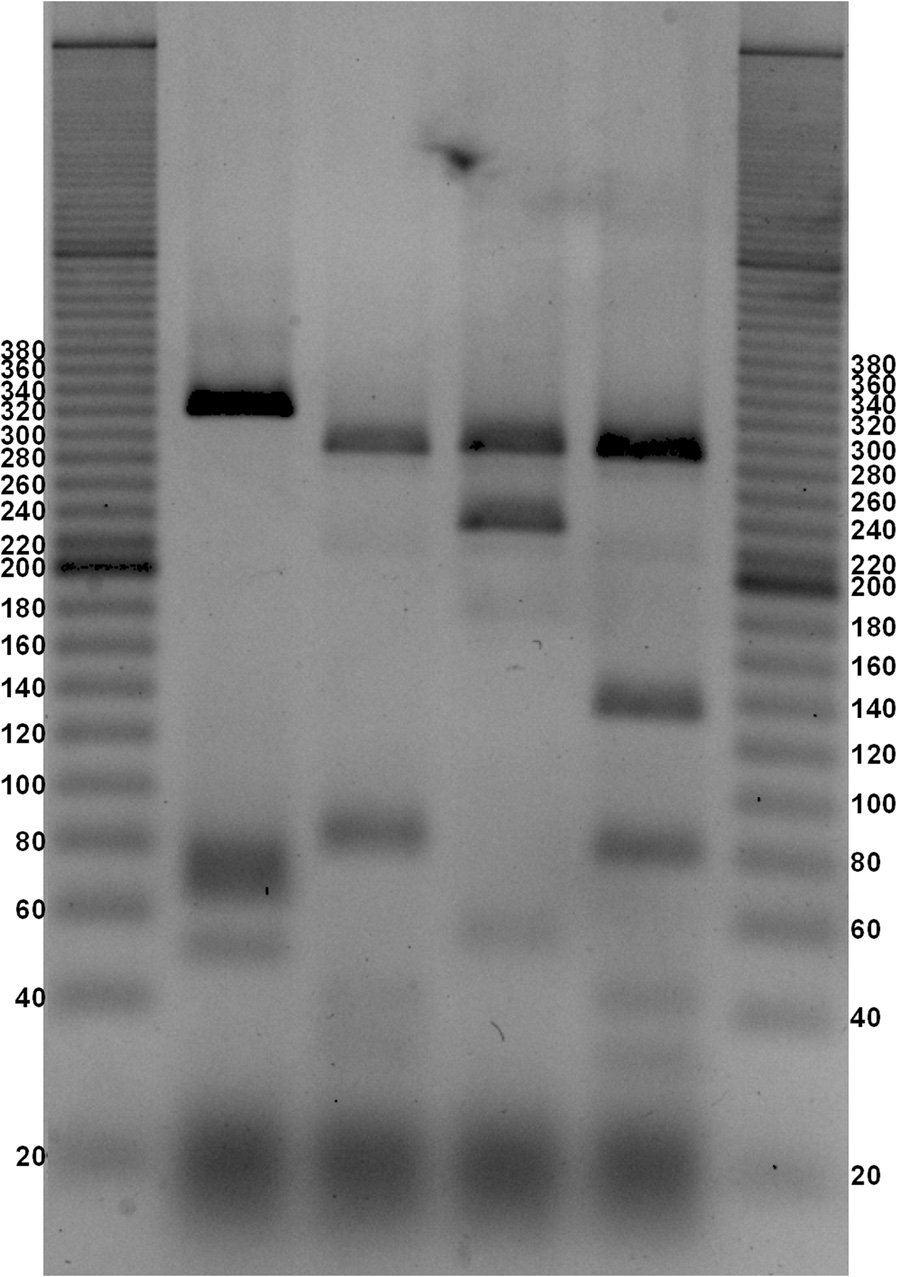
**Agarose gel electrophoresis of digested *hsp60 *DNA fragments with HaeIII (negative image).** Lane1, ladder 20 bp (Sigma-Aldrich); Lane 2, *B. minimum* ATCC 27539; Lane 3, *B. pullorum* ATCC 27685, Lane 4, *B. subtile* ATCC 27537; Lane 5, *B. gallinarum* ATCC 33777; Lane 6, ladder 20 bp (Sigma-Aldrich).

**Figure 3:**
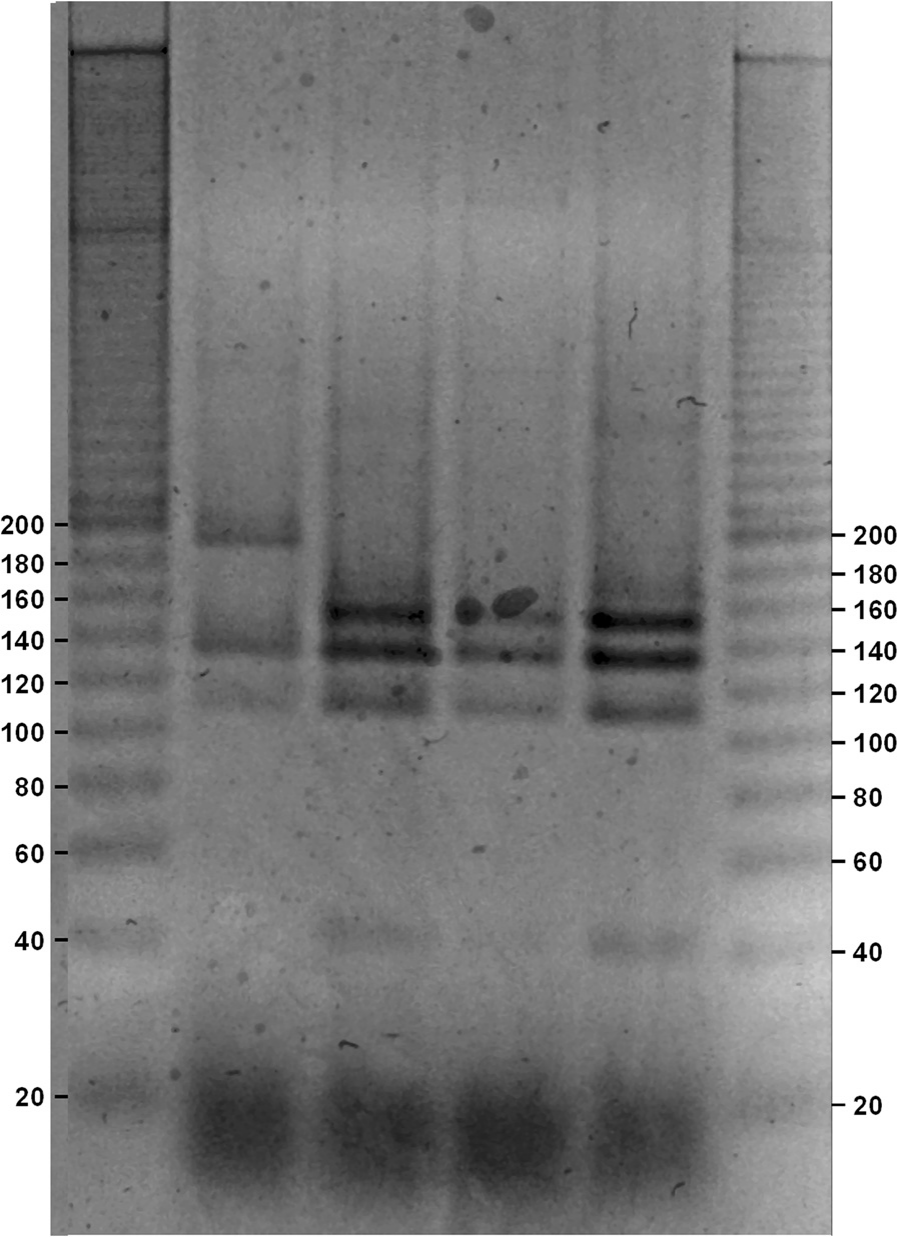
**Agarose gel electrophoresis of digested *hsp60 *DNA fragments with HaeIII (negative image).** Lane1, ladder 20 bp (Sigma-Aldrich); Lane 2, *B. breve* ATCC 15700; Lane 3, *B. longum* subsp. *infantis* ATCC 15697; Lane 4, *B. longum* subsp. *longum* ATCC 15707; Lane 5, *B. longum* subsp. *suis* ATCC 27533; Lane 6, ladder 20 bp (Sigma-Aldrich).

**Figure 4:**
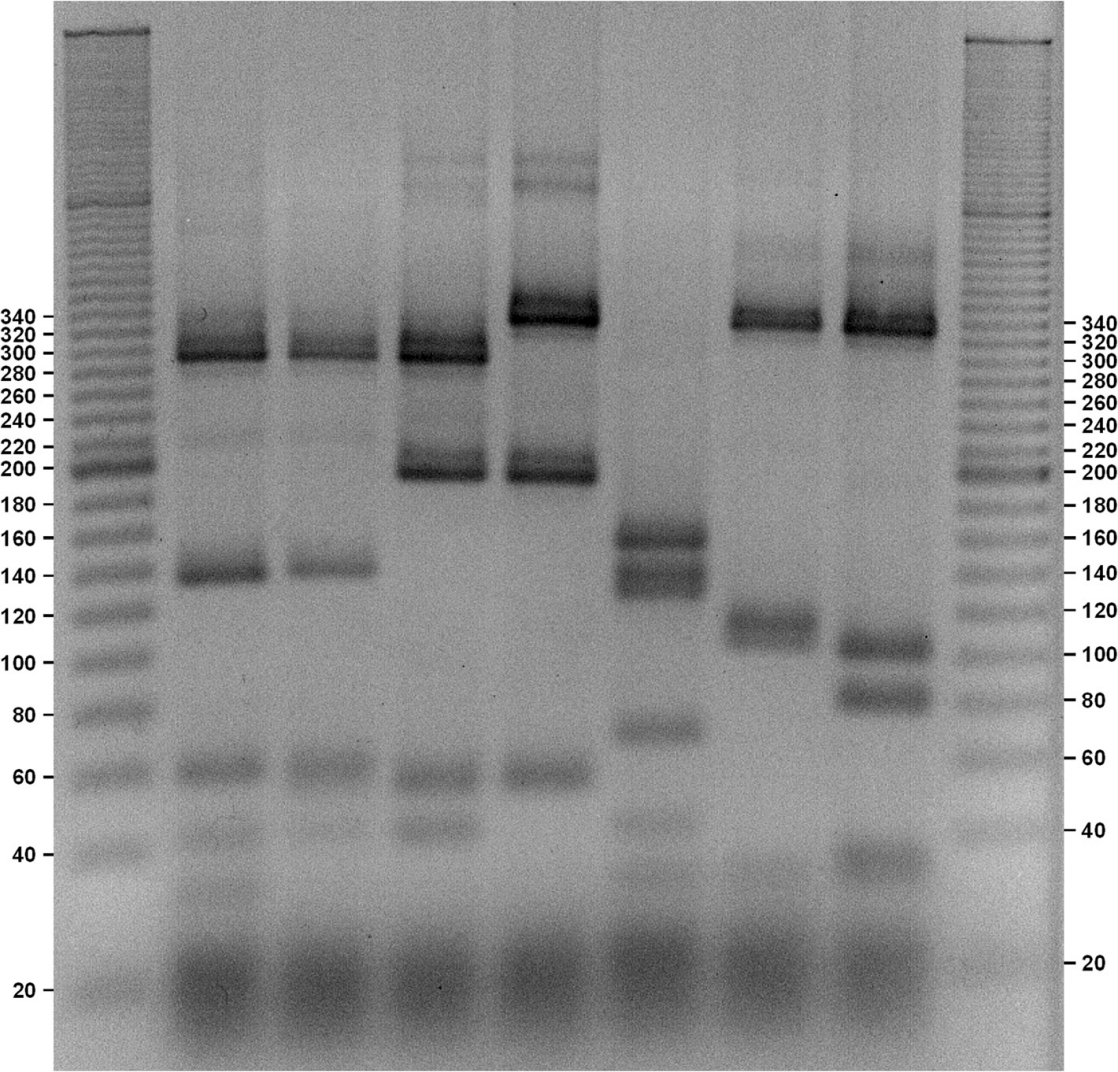
**Agarose gel electrophoresis of digested *hsp60 *DNA fragments with HaeIII (negative image).** Lane1, ladder 20 bp (Sigma-Aldrich); Lane 2, *B. merycicum* ATCC 49391; Lane 3, *B. angulatum* ATCC 27535, Lane 4, *B. pseudocatenulatum* ATCC 27919; Lane 5, *B. catenulatum* ATCC 27539; Lane 6, *B. dentium* ATCC 27534; Lane 7, *B. ruminantium* ATCC 49390; Lane 8, *B. adolescentis* ATCC 15703; Lane 9, ladder 20 bp (Sigma-Aldrich).

**Figure 5:**
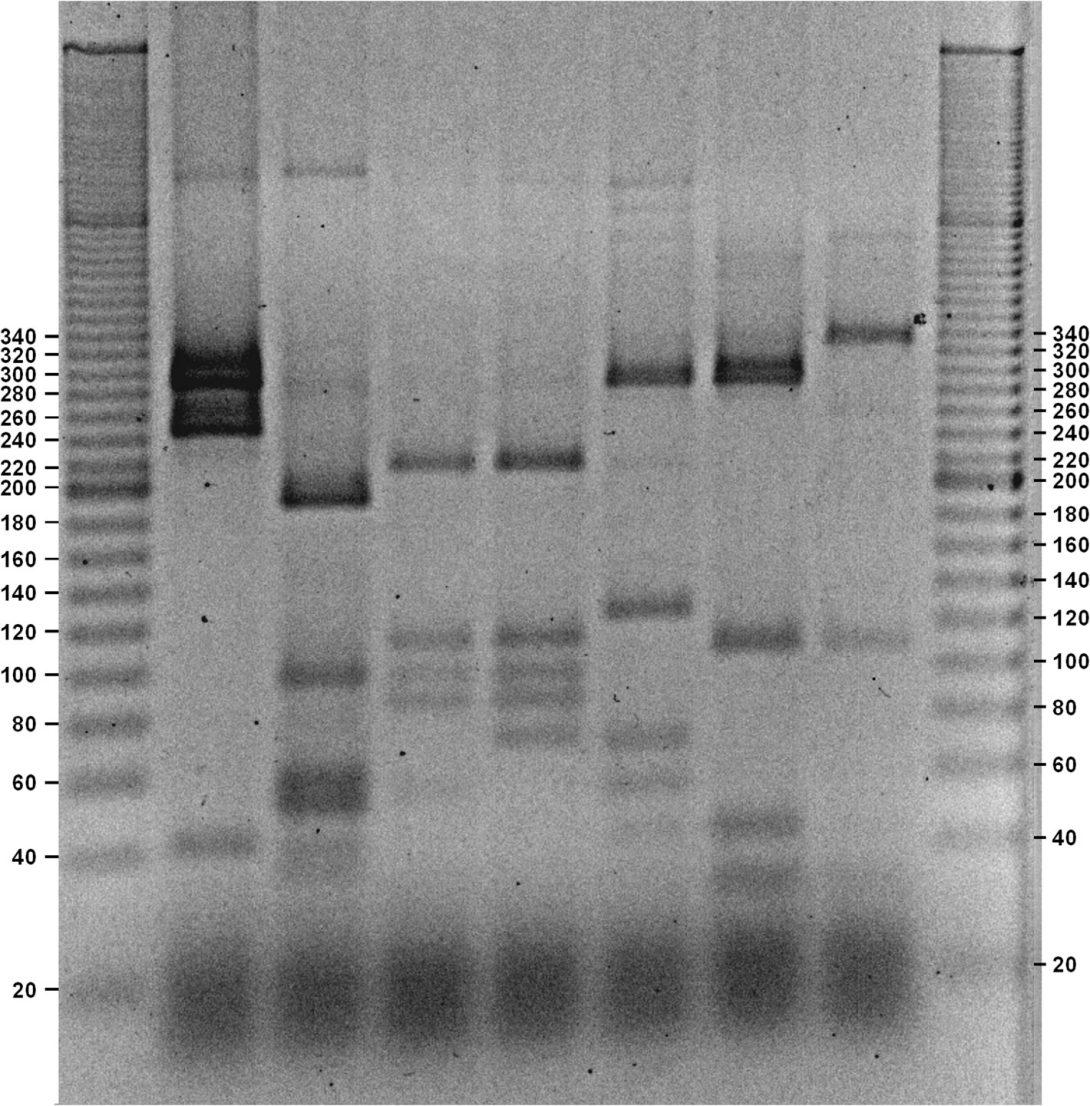
**Agarose gel electrophoresis of digested *hsp60 *DNA fragments with HaeIII (negative image).** Lane1, ladder 20 bp (Sigma-Aldrich); Lane 2, *B. gallicum* ATCC 49850; Lane 3, *B. choerinum* ATCC 27686, Lane 4, *B. animalis* subsp. *lactis* DSM 10140; Lane 5, *B. animalis* subsp. *animalis* ATCC 25527; Lane 6, *B. cuniculi* ATCC 27916; Lane 7, *B. pseudolongum* subsp. *pseudolongum* ATCC 25526; Lane 8, *B. pseudolongum* subsp. *globosum* ATCC 25865; Lane 9, ladder 20 bp (Sigma-Aldrich).

The obtained gels (Figures 1, 2, 3, 4 and 5) show species-specific profiles for all type-strains other than *B. longum* and *B. thermacidophilum* subspecies. This technique does not allow the identification of the subspecies belonging to these species, which displayed identical RFLP profiles. Matsuki et al. [14,17] proposed specific primers to differentiate the subspecies of the species *B. longum*, while *B. thermacidophilum* subsp. *porcinum* and *B. thermacidophilum* subsp. *thermacidophilum* can be differentiated according to Zhu et al. [33]. The proposed restriction analysis is efficient in discriminating very closely related species and subspecies as *B. catenulatum*/*B. pseudocatenulatum*, *B. pseudolongum* subsp. *pseudolongum*/*B. pseudolongum* subsp. *globosum* and *B. animalis* subsp. *animalis*/*B. animalis*. subsp. *lactis*.

The same method has been applied with the use of precast gradient polyacrylamide gels. The resolution was greater than that obtained on agarose gels, loading only 4 μl of the restriction reaction instead of the 30 μl used in horizontal electrophoresis. This may allow to reduce the volume of amplification reactions with a consequent reduction of costs.

**Figure 6:**
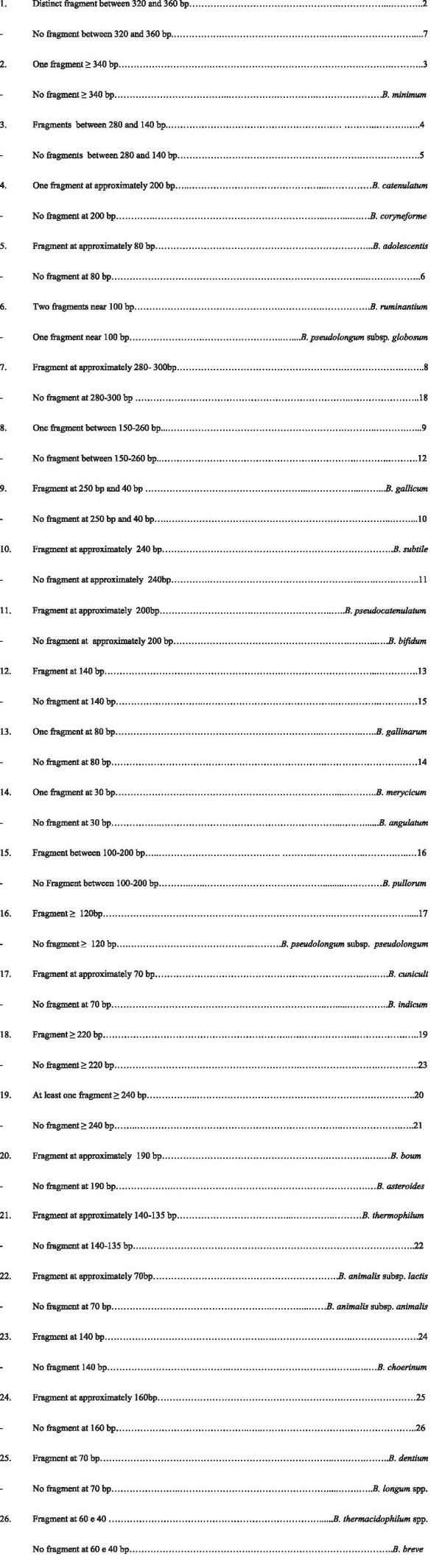
**Dichotomous key to identify species of *Bifidobacterium *based upon HaeIII restriction digestion of ~590 bp of the *hsp60 *gene.**

The comparison between *in silico* digestion and the obtained gel profiles allowed to develop a dichotomous key (Figure 6) for a faster interpretation of the restriction profiles.

### Validation of PCR-RFLP analysis on bifidobacterial isolates

39 strains belonging to 12 different species/subspecies (Table 2) have been investigated to validate the PCR-RFLP technique. Most of the strains tested were previously identified using biochemical tests and in some cases also molecular techniques (species-specific PCR, 16S rDNA sequencing). The obtained data confirmed a conservation of the profiles concerning the species and subspecies tested. Two figures are available as Additional files (Additional file 2: Figure S2: strains belonging to *B. animalis* subsp. *lactis* and *B. animalis* subsp. *animalis*. Additional file 3: Figure S3: strains belonging to *B. longum* subsp. *longum*, *B. longum* subsp. *infantis*, *B. longum* subsp. *suis*). About 95% of the strains confirmed the taxonomic identification previously assigned. Two strains, B1955 and Su864, previously classified as *B. catenulatum* and *B. longum* subsp. *suis* respectively, gave different profiles from those expected. The RFLP profiles of B1955 turned out to be the same of *B. adolescentis* ATCC 15703 (T), the dichotomous key confirmed the assignment to the *B. adolescentis* species. In addition, Su864 was identified as a *B. breve* strain. These results were also verified through a species-specific PCR [14].

## Conclusions

In this work a PCR-RFLP based method to identify *Bifidobacterium* spp. was developed and tested on strains belonging to different species. The technique could efficiently differentiate all the 25 species of *Bifidobacterium* genus and the subspecies belonging to *B. pseudolongum* and *B. animalis*, with the support of an easy-to-handle dichotomous key. The technique turned out to be fast and easy, and presented a potential value for a rapid preliminary identification of bifidobacterial isolates.

## Abbreviations

PCR: Polymerase chain reaction; RFLP-PCR: Restriction fragment length polymorphism; HSP60: Heat-shock protein 60; rDNA: Ribosomal DNA; ARDRA: Amplified ribosomal DNA restriction analysis; DGGE: Denaturing gradient gel electrophoresis; ERIC-PCR: Enterobacterial repetitive intergenic consensus-PCR; RAPD: Random amplified polymorphic DNA; cpnDB: Chaperonin database; TPY medium: Tryptone phytone, yeast medium; BUSCoB: (Bologna University Scardovi Collection of Bifidobacteria).

## Competing interests

The authors declare that they have no competing interests.

## Authors’ contributions

LB conceived the study. LB, VS and ES carried out all the bioinformatics, RFLP analyses, DNA extractions and culture handling. VS conceived the dichotomous key. MM and PM provided some of the strains tested together with the extracted DNA. DDG and FG supervised the work. LB, VS, DDG and FG contributed to paper writing. All authors read and approved the final manuscript. BB supported the research.

## Supplementary Material

Additional file 1: Figure S1 Example of agarose gel electrophoresis of hsp60 amplicons from different bifidobacterial strains. Click here for file

Additional file 2: Figure S2 Agarose gel electrophoresis of digested *hsp60* DNA fragments with HaeIII (negative image). Lane1, ladder 20 bp (Sigma-Aldrich); Lane 2–6, *B. animalis* subsp.*lactis* strains Ra20, Ra18, F439, P23, P32; Lane 7–8, *B. animalis* subsp. *animalis* strains T169, T6/1; Lane 9, ladder 20 bp (Sigma-Aldrich). Click here for file

Additional file 3: Figure S3 Agarose gel electrophoresis of digested *hsp60* DNA fragments with HaeIII (negative image). Lane1, ladder 20 bp (Sigma-Aldrich); Lane 2–4, *B. longum* subsp. *suis* strains Su864, Su908, Su932; Lane 5–6, *B. longum* subsp. *longum* strains PCB133, ATCC 15707 (T); Lane 7–9, *B. longum* subsp. *infantis* strains ATCC 15697 (T), B7740, B7710; Lane 9, ladder 20 bp (Sigma-Aldrich). Click here for file
