# Supplementary figures and images for: Identification of species belonging to the Bifidobacterium genus by PCR-RFLP analysis of a hsp60 gene fragment
Source: BMC Microbiol. 2013 Jul 1;13:149. doi: 10.1186/1471-2180-13-149 (PMC3710250; doi:10.1186/1471-2180-13-149)

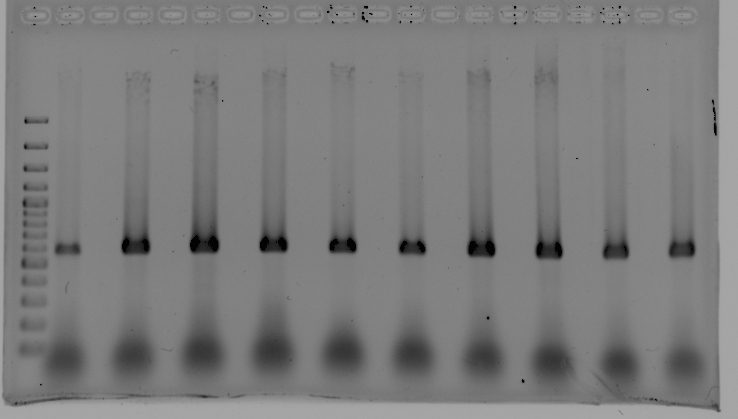

Supplement: Additional file 1: Figure S1 — Example of agarose gel electrophoresis of hsp60 amplicons from different bifidobacterial strains. [file 1471-2180-13-149-S1.jpeg]

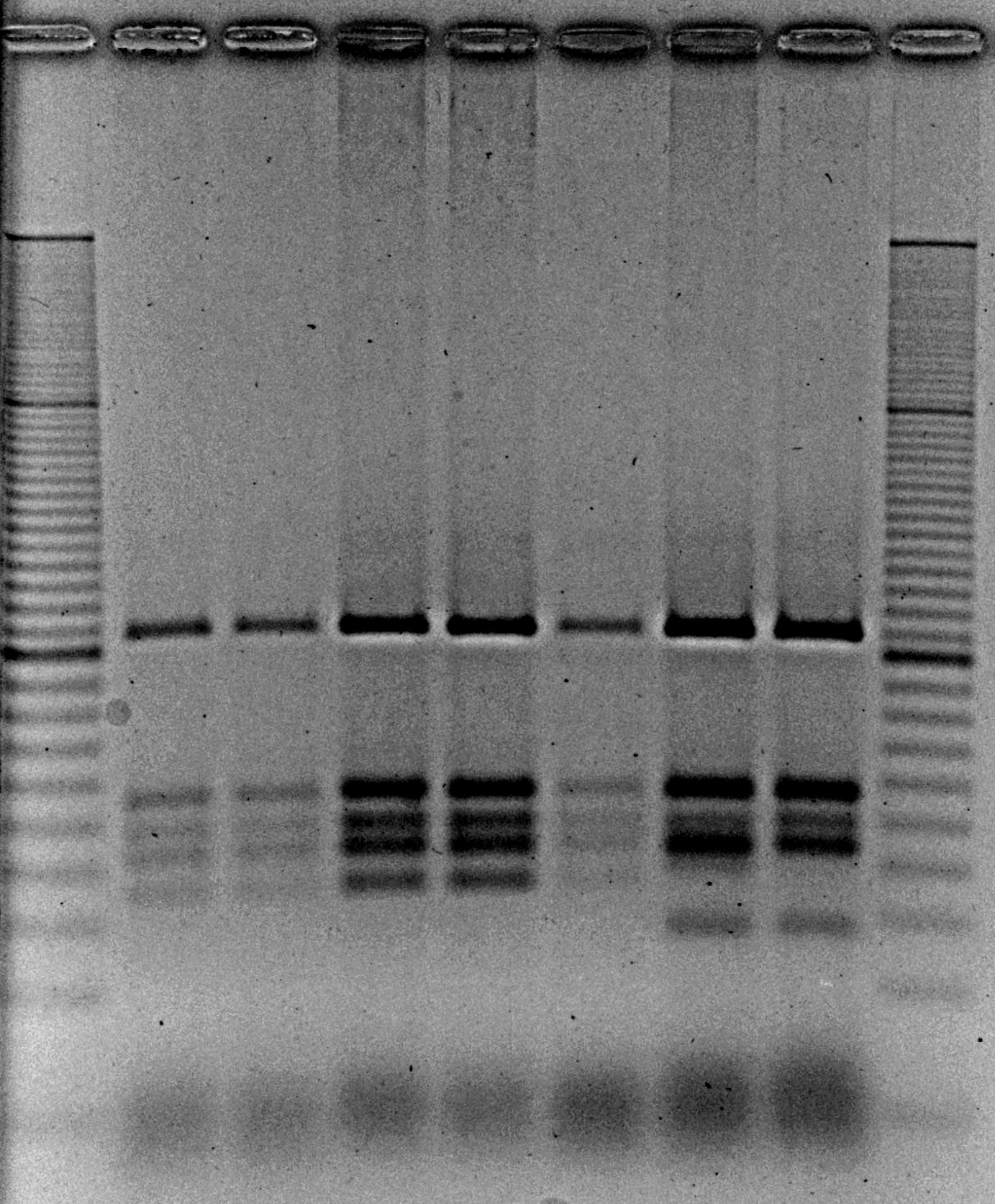

Supplement: Additional file 2: Figure S2 — Agarose gel electrophoresis of digested hsp60 DNA fragments with HaeIII (negative image). Lane1, ladder 20 bp (Sigma-Aldrich); Lane 2–6, B. animalis subsp.lactis strains Ra20, Ra18, F439, P23, P32; Lane 7–8, B. animalis subsp. animalis strains T169, T6/1; Lane 9, ladder 20 bp (Sigma-Aldrich). [file 1471-2180-13-149-S2.jpeg]

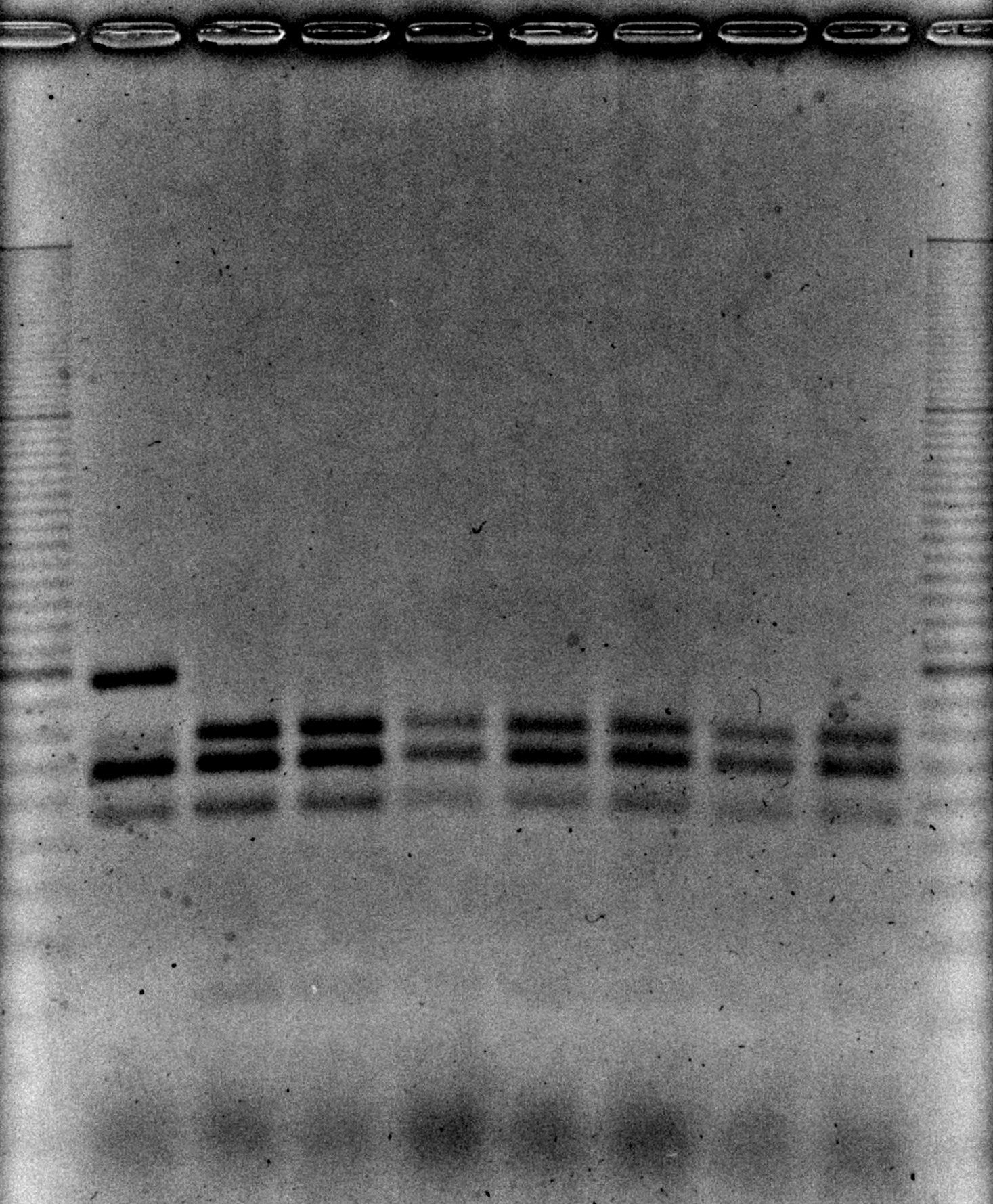

Supplement: Additional file 3: Figure S3 — Agarose gel electrophoresis of digested hsp60 DNA fragments with HaeIII (negative image). Lane1, ladder 20 bp (Sigma-Aldrich); Lane 2–4, B. longum subsp. suis strains Su864, Su908, Su932; Lane 5–6, B. longum subsp. longum strains PCB133, ATCC 15707 (T); Lane 7–9, B. longum subsp. infantis strains ATCC 15697 (T), B7740, B7710; Lane 9, ladder 20 bp (Sigma-Aldrich). [file 1471-2180-13-149-S3.jpeg]
